# 1,5-Anhydroglucitol as a Marker of Acute Hyperglycemia in Cardiovascular Events

**DOI:** 10.1900/RDS.2022.18.68

**Published:** 2022-06-30

**Authors:** Marta Migała, Justyna Chałubińska-Fendler, Marzenna Zielińska

**Affiliations:** 1 Department of Intensive Cardiac Therapy. Medical University of Lodz. Lodz. Poland,; 2 Department of Radiation Oncology. Military Institute of Medicine. Warsaw. Poland.

**Keywords:** acute coronary syndrome, acute hyperglycemia, 1,5-anhydroglucitol, cardiovascular diseases, risk factors

## Abstract

1,5-anhydroglucitol (1,5-AG) is a biomarker of acute hyperglycemia in diabetology and also in cardiodiabetology. It is used to monitor fluctuating glucose levels. 1,5-AG is a monosaccharide that is biochemically similar to D-glucose and originates from the nutrition. The presence of 1,5-AG in blood and tissue is nearly constant due to reabsorption in the renal proximal tubule. In acute hyperglycemia, renal reabsorption is inhibited by glucose and 1,5-AG is excreted in the urine, while its serum level decreases rapidly. 1,5-AG reflects glucose excursions over 1-3 days to 2 weeks. In this regard, low levels of serum 1,5-AG can be a clinical marker of short-term glycemic derangements such as postprandial hyperglycemia, which is an important risk factor for the pathogenesis of coronary artery disease (CAD) as low levels of 1,5-AG reflect severe plaque calcification in CAD and correlate with high-density lipoprotein cholesterol (HDL-C) levels. For these reasons, 1,5-AG may also be a marker for atherosclerosis; in fact an even better marker than HbA1c or fructosamine which are normally used. 1,5-AG may also be a predictor of cardiovascular disease, left ventricular dysfunction after acute coronary syndrome (ACS), and mortality after ACS. This articles reviews the current knowledge on 1,5-AG related to its use as predictor for cardiovascular events.

## 1 Introduction

### 1.1 Markers of acute hyperglycemia of coronary artery disease importance

**Figure 1.**
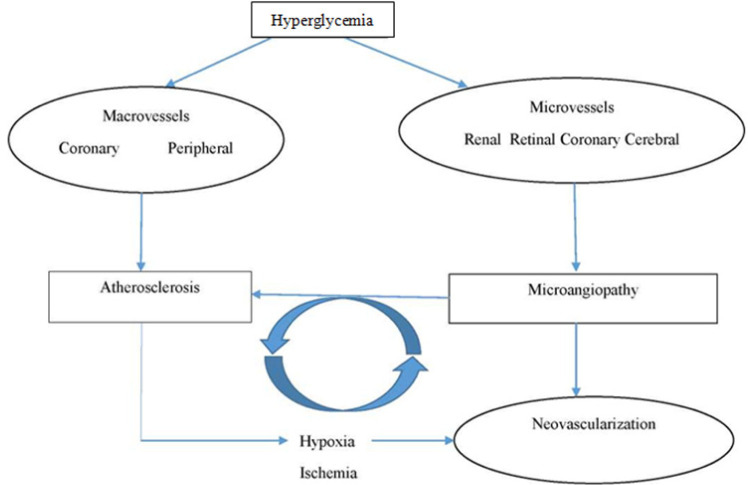
Influence of microangiopathy and macroangiopathy on atherosclerosis [4]. Hyperglycemia is a causative factor for the development of large and small vessel disease. The two pathologies may promote atherosclerosis. In case of microangiopathy, atherosclerosis occurs in the condition of hypoxia. Additionally, microangiopathy and the process of atherosclerosis cause changes in the vasa vasorum and activate neovascularization [4].

There is growing interest in establishing normal and stable glucose levels in patients with coronary artery disease (CAD). It is well known that hyperglycemia is a risk factor for developing CAD, both in diabetic and non-diabetic populations [1]. Abnormal glycemic levels may cause chronic and acute events like acute coronary syndrome or stroke [2,3]. Poor glycemic control may result in microvascular and macrovascular complications (Figure 1) [4]. Therefore, diabetic markers are becoming widely used in monitoring and predicting outcomes in patients with cardiovascular conditions. It is as important to determine glucose levels in patients with CAD using glycated hemoglobin (HbA1c), fructosamine, or fasting blood glucose measurement in non-diabetic patients in the same way as it is done in diabetic patients. However, the measures represent the glycemic status over different time intervals, which must be taken into account when monitoring glycemic risk factors in cardiovascular conditions.

HbA1c reflects glucose levels over a period of 2-3 months, fructosamine those over 2-4 weeks [5,6]. Prolonged exposure to high glucose levels is one of the main causes of microvascular dysfunction (Figure 1) [4]. Therefore, high short-term fluctuations in glucose levels, especially postprandial ones, may be significant in the occurrence of cardiovascular events by inducing endothelial dysfunction, inflammatory reactions, and oxidative stress which may lead to atherosclerosis [7]. In this regard, the assessment of fasting glucose levels alone or in combination with other long-term markers such as HbA1c and fructosamine may be insufficient to monitor the overall risk caused by glycemic derangements. Other complementary markers may be required to obtain information on glycemic status and predict possible cardiovascular complications.

In the long-term observation of patients, HbA1c has become a widely used marker in diabetic and non-diabetic patients. In patients with cardiovascular conditions, it is used to monitor hyperglycemia over 2-3 months, providing information about moderate to severe glucose changes rather than short-term glucose fluctuations [5]. HbA1c is a good reflection of average glucose levels over 2-3 months, and its individual variability is smaller than single glucose measurements. However, there may be inexplicable differences between HbA1c and other measures of mean glycemia, especially in diabetic patients, because of individual, specific glycation and deglycation enzymatic profiles [8]. High levels of HbA1c are associated with significant microvascular complications (renal, retinal, coronary, and cerebral) [9]. Thus, HbA1c was proposed as a marker of higher risk of CAD incidence [10] and related to poor clinical findings in patients with CAD. Sherbiny et al. found that higher HbA1c levels in patients with ST-elevated myocardial infarction (MI) had lower rates of complete revascularization and a higher incidence of adverse cardiac events [11].

Interestingly, Orellana-Barrios et al. found that neither elevated levels of HbA1c nor estimated average glucose levels (eAG) were associated with higher risk of mortality caused by ST-elevated MI [12]. Apart from individual fluctuations in HbA1c, another disadvantage is that HbA1c is a hemoglobin-dependent marker, such that all variations in hemoglobin levels may influence the results. As Hb1Ac levels represent mean glucose concentration over 2-3 months, it is not particularly useful to monitor it in response to treatment regimens because it does not reflect short-term glycemic excursions, i.e. intraday and interday glycemic changes, which impact microvascular and macrovascular complications in particular [13].

Fructosamine (i.e. glucose combined with serum proteins, mainly albumins, but also globulins and lipoproteins) is another marker of interest in patients with impaired glycemic status [14]. It shows moderate to severe fluctuations in serum glucose levels over a shorter period than HbA1c (2-4 weeks) and is a good reflection of average glucose levels during this time. It also reveals fluctuations in plasma proteins [6]. It has been shown that fructosamine and glycated albumin (GA) may be useful to determine the risk of microvascular disease [15]. The main disadvantage of fructosamine use is the fact that high ranges of fluctuations in total protein levels may impair the results of fructosamine concentrations. This may be avoided by assessing GA instead of fructosamine, but in patients with low albumin levels or those treated with albumins the results may still be biased.

In non-diabetic patients with acute cardiovascular events there is a need for an accurate biomarker to assess smaller fluctuations of glucose (mild to moderate levels) with high sensitivity and over short periods of time. A candidate of interest is 1,5-anhydroglucitol which reflects postprandial glycemia. Postprandial glucose levels reflect acute glucose swings and have a triggering effect on oxidative stress in contrast to chronic sustained hyperglycemia [16].

## 2 Methods

### 2.1 1,5-AG as a marker of acute hyperglycemia and its usefulness

1,5-anhydroglucitol in serum is a short-term marker of glycemic control and appears to be the most suitable parameter for monitoring glucose excursion. As discussed above, fasting plasma glucose, fructosamine, and HbA1c have limitations in measuring daily glucose excursion [17]. HbA1c measurements reflect blood glucose over the past 2-3 months, while fructosamine can be used to evaluate glycemic control over 2-4 weeks. Random plasma glucose assesses only actual glucose level. 1,5-AG levels in blood have the advantage of reflecting glucose levels over the last 24-48 hours [18]. Thus, serum levels of 1,5-AG can reflect short-term glucose excursion and postprandial hyperglycemia more sensitively than HbA1c in subjects with moderately controlled diabetes mellitus or impaired glucose tolerance (IGT) [19,20].

Stettler et al. suggested that 1,5-AG may be complementary to HbA1c and fructosamine measurements in evaluation of glycemic control [21-23]. 1,5-AG may also be helpful in monitoring short-term glycemic changes in preoperation, preconception, pregnancy, and after glycaemia-related therapeutic changes, and it may be capable of detecting risk factors for the development of macrovascular complications such as cardiovascular disease [24,25]. Postprandial hyperglycemia is an important risk factor for the pathogenesis of CAD and cardiovascular events, even death [26].

The Diabetes Epidemiology Collaborative Analysis of Diagnostic Criteria in Europe (DECODE) study showed that postprandial hyperglycemia was a better predictor of mortality in patients with overt diabetes mellitus and in those with impaired glucose tolerance than fasting glucose [27]. Dungan indicated that in patients with acute coronary syndrome, 1,5-AG may be useful to distinguish subjects with stress hyperglycemia from normoglycemic subjects [28]. Watanabe et al. discovered that lower levels of serum 1,5-AG are useful to detect men at higher risk of cardiovascular disease regardless of the presence or absence of diabetes [29]. No significant relationship between serum 1,5-AG levels and the risk of cardiovascular disease was observed in women [29].

## 3 Results

### 3.1 Diagnostic and prognostic value of low levels of serum 1,5-anhydroglucitol on cardiovascular disease

Ito et al. showed that 1,5-AG levels were significantly lower in patients with acute coronary syndrome (ACS) among non-diabetic patients than those with effort angina pectoris. They also discovered that 1,5-AG correlated with HDL-C, which has substantial impact on the antiatherogenic process. Thus, 1,5-AG could be an additional marker to be used in association with common and glycemic risk markers in patients with ACS [30].

Fujiwara et al. observed that lower 1,5-AG is associated with CAD, even in controlled diabetic patients and non-diabetics [31], suggesting that 1,5-AG is a useful marker for detecting patients with postprandial hyperglycemia at high cardiovascular risk, even non-diabetic patients. While other studies showed that HbA1c is significantly associated with coronary artery disease in diabetic and non-diabetic patients [32,33]. 1,5-AG was an even better marker of CAD than HbA1c or GA in the Fujiwara study [31]. In another study, Fujiwara et al. observed that in non-diabetic patients lower 1,5-AG was associated with any coronary revascularization and target lesion revascularization (TLR) present and may help to predict adverse clinical events after percutaneous angioplasty [34].

Selvin et al. showed that in persons without a diagnosis of diabetes distribution of 1,5-AG is nearly normal and concluded that the level of 1,5-AG should be interpreted in relation to HbA1c or fasting glucose. In patients with diagnosed diabetes, distribution of 1,5-AG was lower. Low values for 1,5-AG (<6 ug/ml) were strongly associated with CAD, ischemic stroke, heart failure, and death compared with subjects with >6 ug/ml. Therefore, 1,5-AG has a prognostic value for long-term complications in the diabetic subjects [35].

Ouchi et al. discovered that low levels of 1,5-AG predict long-term cardiac mortality (70 months) in ACS patients with HbA1c levels <7%. The cut-off point was the median level of 1,5-AG (18,5 ug/ml) [27]. Takahashi was the first to report serum 1,5-AG levels to be significantly associated with adverse clinical events in CAD, indicating postprandial hyperglycemia to be an important risk factor for cardiovascular disease in patients with HbA1c levels <7% following first elective percutaneous coronary intervention PCI [36].

Wada et al. showed that low 1,5-AG was associated with more severe plaque calcification, as detected by intravascular ultrasound (IVUS) imaging, which indicated plaque atherosclerosis [37]. Therefore, 1,5-AG may be a useful marker to predict the incidence of severe coronary calcification and future cardiovascular events. It is worth noting that, in this study, coronary atheroma volume and plaque burden were not significantly associated with serum levels of 1,5-AG [37]. A recent study conducted by Su et al. confirmed that 1,5-AG serum levels are associated with high risk of coronary plaque rupture in diabetic patients with ACS, which correlates with postprandial glycemia and is related to the pathogenesis of plaque rupture in diabetes [38].

Torimoto et al. discovered that vascular endothelial function, evaluated by peripheral vascular arterial tonometry (PAT), correlated robustly with 1,5-AG, indicating that the level of 1,5-AG is a useful predictor of vascular endothelial function in patients with HbA1c < 8% and that treatment of postprandial hyperglycemia and atherosclerosis should be provided to patients with low 1,5-AG levels [39].

Ishida et al. demonstrated that low plasma 1,5-AG was closely associated with the extent of severely injured myocardium and systolic and diastolic function in patients at first month after acute myocardial infarction (AMI), suggesting that postprandial glycemia may become a new predictor of left ventricular dysfunction after AMI [40].

## 4 Discussion

### 4.1 Structure and history of 1,5-anhydroglucitol

**Figure 2.**
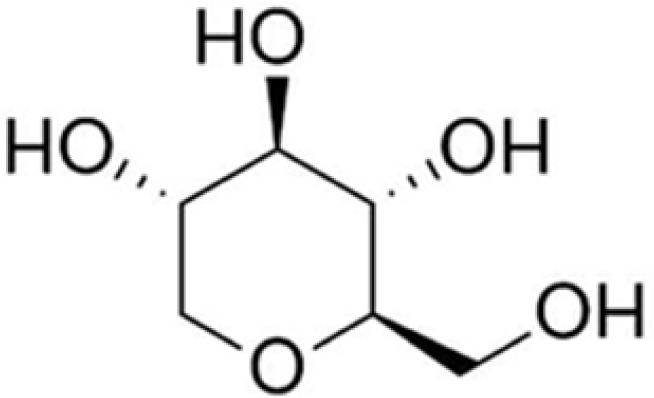
Structure of 1,5 AG

1,5-anhydroglucitol (1,5-AG) is a 6-carbon monosaccharide (polyol), similar to D-glucose, which occurs naturally in human plasma [18]. The structure and physical characteristics are shown in Figure 2.

1,5-AG was isolated from Polygala senega for the first time in 1888 [23,25]. The chemical structure was defined in 1943. In 1972, Pitkanen discovered 1,5-AG in human plasma and in 1973 in cerebrospinal fluid [23, 25]. The first commercial product was successfully created in Japan in 1991 [25]. In 2003, the FDA approved the Glycomark^TM^ test for monitoring 1,5-AG as a marker of short-term glycemic control [25,28].

### 4.2 Dietary sources and metabolism of 1,5-AG

1,5-AG occurs physiologically in all organs and tissues [25], and the human body is estimated to contain 500-1000 mg [41]. Daily intake is about 4.5 mg/day [41]. There is minimal daily fluctuation in concentrations, mainly because 1,5-AG takes part in metabolic processes to a minor extent only, and its intake is similar in quantity to excretion [41]. In healthy subjects, the level of 1,5-AG in serum varies widely (12-40 ug/ml) with higher levels in males than females [42].

The main source of 1,5-AG is soya, while small quantities of 1,5-AG are also found in rice, pasta, meat, fish, fruits, vegetables, tea, milk, and cheese [28]. It is considered that normal diets have no impact on 1,5-AG concentrations. Some studies suggest that Asians and Africans have significantly higher mean baseline levels than Caucasians [22].

1,5-AG levels are relatively lower in Japanese than in Americans [28,43]. 1,5-AG is absorbed in the intestine and excreted in the kidney where it is reabsorbed at 99.9% by active sodium-glucose co transporter (SGLT4), which is localized in both the intestine and proximal renal tubules [25,44]. Only a small amount of 1,5-AG, about 0,5 mg/day, is synthetized de novo supposedly in the liver [25]. Half-life is estimated at about 1-2 weeks [23, 28].

The most characteristic feature is that it is metabolically inert, which means that the 1,5-AG eaten with food is balanced by the portion eliminated by the kidneys [25]. Thus, there is a stable concentration of 1,5-AG, which is not affected by prandial state, weight, or age [25]. When serum glucose level exceeds the renal glucose threshold (typically >180 mg/dl), tubular reabsorption of 1,5-AG is blocked by SGLT4, causing increased urinary loss and reduced serum concentration [28,45,46]. Some studies have reported that 1,5-AG could reflect glucose excursion over a short period of time ranging from 1 to 3 days and 2 weeks [22,28,47]. However, the regeneration of 1,5-AG resources remains slow (about 0.3 ug/ml) and could take as much as 5 weeks [24,28,48].

### 4.3 Assessment methods of 1,5-AG in serum, urine, and cerebrospinal fluid

1,5-AG levels can be measured in serum, plasma, cerebrospinal fluid or in urine [25]. In serum, 1,5-AG is stable at 2-8 C° for 7 days and at 22 C° for 5 days [25]. Samples of fluids can be frozen for long-term storage at -80 C° [25]. Sample shipment, if necessary, should be made in ice or in dry ice in case of long-distance shipping [25]. 1,5-AG in serum is measured by enzymatic kits, gas chromatography mass spectrometry (GC/MS), or high-performance liquid chromatography (HPLC). Its blood serum levels in adults range from 12-40 μg/ml [42].

The reference method for assessment of 1,5-AG is HPLC. However, enzymatic methods are useful as well since they are more convenient and less laborious [49,50].

There are two enzyme assays for determining 1,5-AG in the blood: Glyco Mark^TM^ (Glyco Mark, Inc) approved in USA and Determiner L (Kyowa Medex, Tokyo) used in Japan [51]. These two assays are comparable and can be utilized interchangeably [51]. Enzymatic methods can be used in automated chemistry analyzers such us Hitachi 917 [52].

The recommended volume of serum is 1 ml of 1,5-AG concentration and fasting is not required [25]. Alternatively, 1,5-AG in serum may be measured using chromatography techniques. These methods are sensitive and precise, but time-consuming and cumbersome [28]. Analyzing 1,5-AG by GC/MS requires labor-intensive preparation of the sample by derivatization typically via acetylating processes [53]. HPLC analysis of 1,5-AG is determined by passing the samples through a 2- or 3-layer ion exchange column and subsequent analysis by HPLC with pulsed amperometric detection or enzyme sensor. Typical detection limits for 1,5-AG are reported as being 100-200 ng/ml [53]. For the GlycoMark test, the reference range of 1,5-AG is between 10 and 31 μg/ml [54].

A more sensitive method is needed to analyze 1,5-AG in urine, because its level is significantly lower than in plasma. The detection limit of 1,5-AG in urine is as low as 0.06 μg/ml in 0.1-0.2 ml of urine [53]. Liquid chromatography and ion trap mass spectrometry (LC/ MS3) assay may be a sensitive and selective method of determining 1,5-AG in urine as well. This method provides sufficient selectivity and sensitivity for analysis in 50 μl human urine [53].

### 4.4 Conditions affecting serum levels of 1,5-AG

To consider the usefulness of 1,5-AG in the assessment of glucose fluctuations it is necessary to keep in mind that some subgroups of patients may have alterations in 1,5-AG. It is assumed that, in patients with end-stage chronic kidney disease (stages 4-6), the reabsorption of 1,5-AG is decreased because of the reduction in SGLT4 and aggravated damage of glucose co transporters, but there is no actual data to confirm this hypothesis to date [55].

In mild or moderate renal dysfunction (stages 1-3), 1,5-AG levels reliably reflect glycemic state and may be used in evaluating glycemic control in these patients [55]. Other groups of patients may exhibit decreased level of 1,5-AG as well, namely those after gastrectomy and those under steroid therapy [24].

### 4.4 Clinical utility of 1,5-AG in cardiology

There is no clear recommendation in favor of the use of 1,5-AG in cardiology, especially in non-diabetic patients. To avoid mistakes in this group, results must be interpreted in relation to HbA1c or fasting plasma glucose. Some studies indicate that 1,5-AG is associated with atherosclerotic disease, including coronary artery disease and the risk of revascularization after elective percutaneous coronary intervention, but the enrolled subjects were diabetic as well [36,56,57]. Only two studies suggest that low levels of 1,5-AG are a risk factor for cardiovascular disease and predict long-term cardiac mortality in ACS in patients with HbA1c <7.0% [27]. Other publications confirm that low levels of 1,5-AG are strongly and independently associated with cardiovascular outcomes and mortality, but these observations were also made in patients with diabetes [35].

Thus, it is worth investigating the role of 1,5-AG in patients with cardiovascular disease without diabetes as 1,5-AG is considered to be a marker with high diagnostic and prognostic potential in cardiology. Despite the fact that 1,5-AG is a valuable parameter of glycemic control, it is not commonly used. One of the reasons is probably racial variability relating to higher baseline mean values of 1,5-AG in Asians and Africans than in Caucasians [22] and lower levels in Japanese than Americans [28,43]. It was primarily the Japanese who introduced it to clinical practice in 1991 and used it for at least 20 years [42,58].

Beside its restricted use, another limitation is the lack of established correlations between 1,5-AG cutoffs in microvascular and macrovascular complications of hyperglycemia [59]. Moreover, there is lack of large cohort studies confirming the utility of 1,5-AG on a large scale. Hence, further studies are necessary to provide evidence of better glycemic control and predict cardiovascular risk.

## 5 Conclusions

1,5-AG is a sensitive marker of postprandial hyperglycemia and may be complementary to HbA1c and fructosamine measurements in assessment of glycemic control. 1,5 AG may be helpful in monitoring short-term glycemic changes in preoperation, preconception, pregnancy and after glycemia-related therapeutic changes. In cardiodiabetology low levels of 1,5-AG are associated with increased risk of macrovascular complications such as coronary artery disease throughout severe coronary calcification or cardiovascular events, even deaths. In addition, 1,5-AG may also predict long-term cardiac mortality predominantly in patients with diabetes and in some studies in non-diabetic subjects as well. Despite its significance, 1,5-AG has limitations. One of the reasons why 1,5-AG is not commonly used is racial variability in higher baseline mean values of 1,5-AG and lack of established 1,5-AG cut-offs in microvascular and macrovascular complications. Finally, no large cohort clinical studies have been conducted to confirm the utility of 1,5-AG for a general use. Future research should be done to standardize the level of 1,5- AG and predict cardiovascular complications.

## Abbreviations

1,5-AG: 1,5-anhydroglucitol

ACS: acute coronary syndrome

AMI: acute myocardial infarction

CAD: coronary artery disease

DECODE: Diabetes Epidemiology: Collaborative Analysis of Diagnostic Criteria in Europe

GA: glycated albumin

HPLC: high performance liquid chromatography

HbA1c: glycated hemoglobin

IVUS: intracoronary ultrasound

PAT: peripheral vascular arterial tonometry

TLR: target lesion revascularization
